# Supplementary material for: Differential bioenergetic profile of human glioblastoma following transplantation of myocyte-derived mitochondria
Source: PLoS One. 2025 Oct 14;20(10):e0330322. doi: 10.1371/journal.pone.0330322 (PMC12520375; doi:10.1371/journal.pone.0330322)
Supplement: S1 File — All data confirm the correct parental lineage. (PDF) [file pone.0330322.s001.pdf]

LOCUS	**U3046**		**U3046-PCS**		**U3035**		**U3035PCS**	
3S1358	14	18	14	18	16	17	16	17
TH01	9	9.3	9	9.3	9		9	
D21S11	28	31	28	31	28	30.2	28	30.2
D18S51	14		14		14	18	14	18
Penta_E	10	11	10	11	10	11	11	
D5S818	12		12		10		10	
D13S317	9		9		12		12	
D7S820	10	12	10	12	10		10	
D16S539	11		11		11	14	11	14
CSF1PO	10	12	10	12	12		12	
Penta_D	9	15	9	15	12	13	12	13
Amelogenin	X	Y	X	Y	X		X	
vWA	17	19	17	19	16	18	16	18
D8S1179	13	15	13	15	11	12	11	12
TPOX	8	11	8	11	8	11	8	11
FGA	23	25	23	25	21	25	21	25
D19S433	13	14	13	14	14	15	14	15
D2S1338	20	22	20	22	17		17	
