# Supplementary material for: Differential bioenergetic profile of human glioblastoma following transplantation of myocyte-derived mitochondria
Source: PLoS One. 2025 Oct 14;20(10):e0330322. doi: 10.1371/journal.pone.0330322 (PMC12520375; doi:10.1371/journal.pone.0330322)
Supplement: Attachment — Submitted filename: PONE-D-25-41544_Response_to_reviewers_v1.pdf [file pone.0330322.s003.pdf]

Responses to Reviewers (**PONE-D-25-41544)**

Reviewer #1:

1.The resolution of all figures need to be improved.

Thank you for bringing the resolution to our attention. All figures have been formatted according to the publisher's requirements using TIFF format and 600 dpi.

2. The signal of Figure 1-B is weak, seems like background, please improve the quality of picture.

This figure has been formatted to 600 dpi and the mitochondrial dye is more clearly visible. Once again, this figure represents the baseline mitochondrial content of the cells prior to mitochondrial transplant from the myocyte donor cells, so we anticipated that the intensity of staining would be less than the red tracker dye (myocyte only) and the overlay (native; green and donor; red).

3. For Figure 3A, please show the conducting statistics.

A table has been inserted into this figure with the time specific values and standard deviations for both the OCR and ECAR data.

Reviewer #2: *The well written and coherent work describes research on mitochondrial transplantation using non-neoplastic, human myocyte-derived mitochondria into two mesenchymal subtype glioblastoma cell lines. The study investigates the impact on metabolic architecture and response to ionizing radiation, combining functional bioenergetic assays and transcriptomic profiling. The work is well presented, conceptualized and scientifically sound. I have several points that could help to improve the already great study.*

Questions:

Can the authors comment on the purity and integrity of the isolated mitochondria after transplantation?

The mitochondrial isolation was performed using a commercially available kit with manufacturer provided technical references. Although we performed the JC-1 assay on the transplants under stress and non-stress conditions, no additional test of mitochondrial function or membrane integrity was performed. We can assume that the response in the U3035 cells indicates that functional mitochondria and the U3046 cells received the same complement of donor mitochondria from the same extractions. We have mentioned this limitation to the process in our revised discussion:

Discussion Page 15, Line 23:

> "Our work also indicates that transplanted mitochondria maintain polarization even under nutrient-limited or hypoxic conditions and reduced radiation-induced depolarization, suggesting a stabilizing effect on mitochondrial integrity under metabolic stress."

A possible limitation is the short post-translation effect (48 hours), could long-term studies improve the understanding of therapeutic durability?

In our previous work on this topic, we employed the strategy of mitochondrial transplantation from one distinct glioblastoma cell line to another, using the same donor: recipient dye combinations (Marshall et al, 2025). In that study, we assayed for the retention of donor mtDNA in recipient cells up to 14 days post-transplantation. Within the revised discussion, we review the potential applications for mitochondrial transplantation largely encompassing adjuvant therapy to the standard of care given in a relatively short interval. We have mentioned the need for "long-term" studies in the discussion and how these would benefit translational potential:

Discussion Page 17 Line 14:

> "Rather than a direct cytotoxic strategy, mitochondrial augmentation may represent a precision therapeutic approach to unmask latent metabolic liabilities in adaptable GBM subtypes. Stratification based on baseline metabolic engagement and radiation transcriptional profiles may help identify patients most likely to benefit from metabolism- and redox-directed combination strategies. This builds on our prior work that significant heterogeneity in response exists among glioblastoma cell lines, even those that share mesenchymal gene expression profiles [24]. Further *in vivo* studies will aid in elucidating the impact that mitochondrial transplantation could have in optimizing standard of care treatment for glioblastoma as a therapeutic adjunct."

In addition, the observation that U3046 cells show higher mitochondrial uptake but less functional augmentation raises questions about mitochondrial turnover, integration, or degradation in different subtypes.

Thank you for addressing this phenomenon. One of our central conclusions to this study is the potential variable response to mitochondrial transplantation based on the recipient cell line. This underscores the heterogeneity of glioblastoma that becomes apparent when standard treatments, such as radiotherapy, are undertaken. We have discussed this in one of our other previous studies (Cifarelli et al, 2021) and have incorporated it into the revision:

Discussion: Page 15, Line 22:

> "Notably, U3046 cells exhibited more persistent mitochondrial signal post-transplantation, possibly reflecting enhanced uptake or reduced turnover. Our work also indicates that transplanted mitochondria maintain polarization even under nutrient-limited or hypoxic conditions and reduced radiation-induced depolarization, suggesting a stabilizing effect on mitochondrial integrity under metabolic stress."

Discussion: Page 17, Line 16:

> "Stratification based on baseline metabolic engagement and radiation transcriptional profiles may help identify patients most likely to benefit from metabolism- and redox-directed combination strategies. This builds on our prior work that significant heterogeneity in response exists among glioblastoma cell lines, even those that share mesenchymal gene expression profiles [24]."

There is minimal discussion on whether mitochondrial transplantation could inadvertently support tumor growth in some contexts or induce off-target effects.

This was an initial concern of ours as well and emphasizes the need for further *in vivo* studies. We have discussed this in the limitations section:

Discussion Page 17 Line 3:

> "While promising, this study has important limitations. Chief among them is the absence of in vivo outcome data and the lack of assessment regarding how mitochondrial transplantation may influence the long-term proliferative capacity of GBM cells. Addressing the former would require rodent model testing, which is complicated by the challenge of selecting a representative cell line—especially given the significant functional differences we observed between just two human GBM lines. Moreover, the increasing recognition of immune system involvement in GBM response would necessitate either the use of rodent glioma lines or humanized models, both of which may limit translatability. Regarding the potential impact on proliferation, our prior study using neoplastic donor and recipient cells did not demonstrate any increase in proliferative index [7]. However, future investigations—particularly those involving different donor:recipient combinations—will need to closely evaluate this possibility."

The use of the cell-penetrating peptide Pep-1 is described, but the efficiency, potential toxicity, and specificity of this delivery mechanism are not deeply discussed.

CPPs, including Pep-, have been used in clinical models. We have updated the discussion with additional references:

Discussion Page 15, Line 18:

> "Using a Pep-1 peptide–mediated delivery platform, we achieved efficient mitochondrial transfer from skeletal myocytes, confirmed by fluorescent signal tracking. CPPs, such as Pep-1 have a substantial amount of data supporting the efficiency of transport into a donor cell [18, 19]. Moreover, recent studies have confirmed that this approach can be clinically translatable with no evidence of toxicity[20]."

Clarification on statistical methods and whether all replicates are biological or technical would clarify the validity of the findings.

These notes are made within the figure captions for each data figure where applicable.

The authors should address how the findings of the work could translate into in vivo models

Discussion Page 17, Line 14:

> "Rather than a direct cytotoxic strategy, mitochondrial augmentation may represent a precision therapeutic approach to unmask latent metabolic liabilities in adaptable GBM subtypes. Stratification based on baseline metabolic engagement and radiation transcriptional profiles may help identify patients most likely to benefit from metabolism- and redox-directed combination strategies. This builds on our prior work that significant heterogeneity in response exists among glioblastoma cell lines, even those that share mesenchymal gene expression profiles [24]. Further in vivo studies will aid in elucidating the impact that mitochondrial transplantation could have in optimizing standard of care treatment for glioblastoma as a therapeutic adjunct."

Minor comments:

In methods, the composition of mitochondrial isolation buffer should be given

The mitochondrial isolation is performed using a commercially-available kit (BioVision; K288-50), now Abcam; ab288084). The manufacturer does not provide the composition of this proprietary buffer.

Beginning of discussion: “granualr" is mistyped

Corrected

The picture depicted in fig 6a was created de novo by authors, or adopted from different work? If the latter is true, it should be referenced. The same applies for 6b.

Thank you for identifying this oversight on our behalf. The figures from the KEGG pathways have been granted copyright release from the appropriate owners and documentation of such as been provided.
