# Supplementary material for: Differential bioenergetic profile of human glioblastoma following transplantation of myocyte-derived mitochondria
Source: PLoS One. 2025 Oct 14;20(10):e0330322. doi: 10.1371/journal.pone.0330322 (PMC12520375; doi:10.1371/journal.pone.0330322)
Supplement: Attachment — Submitted filename: PONE-D-25-41544_Response_to_reviewers_v2.docx [file pone.0330322.s004.docx]

Responses to Reviewers (**PONE-D-25-41544R1)**

*Reviewer #1:*

*1. The quality of Figure 5 is not good. It is hard to identify the letter. Please improve it.*

Figure 5 has been re-uploaded at 1200dpi, which exceeds the requirements of the Journal.

*2. The quality of Figure 4A, 4B are not good. The letter of the top is hard to identify.*

Figures 4A and 4B has been re-uploaded at 1200dpi, which exceeds the requirements of the Journal.

*3. Please reorganize the order of all figures.*

The order has been re-checked and is in accordance with the text.

*Reviewer #2: The authors answered all my questions and addressed the concerns. I recommend the work for publication and congratulate them on the great work.*

Thank you for your support.
