# Supplementary material for: Differential bioenergetic profile of human glioblastoma following transplantation of myocyte-derived mitochondria
Source: PLoS One. 2025 Oct 14;20(10):e0330322. doi: 10.1371/journal.pone.0330322 (PMC12520375; doi:10.1371/journal.pone.0330322)
Supplement: Attachment — Submitted filename: PONE-D-25-41544R2_Response_to_reviewers.docx [file pone.0330322.s005.docx]

Responses to Reviewer (**PONE-D-25-41544R2)**

*Reviewer #1: Reviewer #1: It looks fine now. I agree to publish it. But the quality of Figure 5 is not good. It is better to improve the resolution of it.*

Figure 5 has been revised again.
